# Studies on Food Physical Characterization

**DOI:** 10.3390/foods13101572

**Published:** 2024-05-17

**Authors:** Lubomír Lapčík

**Affiliations:** 1 Department of Foodstuff Technology, Faculty of Technology, Tomas Bata University in Zlín, Nam. T.G. Masaryka 5555, Zlín 760 01, Czech Republic; lapcikl@seznam.cz; 2 Department of Physical Chemistry, Faculty of Science, Palacky University in Olomouc, 17. Listopadu 12, Olomouc 771 46, Czech Republic

With the growing focus on sustainable food production, there has been a surge in research aimed at developing innovative and eco-friendly food alternatives. This trend has led to an influx of innovative products flooding the market, catering to diverse dietary preferences and sustainability concerns. Notable among these are plant-based alternatives like vegan cheese, milk, and high-protein snack bars, which have garnered considerable attention from consumers and researchers alike. The rise of these products has spurred interest in exploring a wide array of plant-based sources, ranging from lentils and chickpeas to wheat bran and beyond. Researchers are delving into the potential of these ingredients not only for their nutritional value but also for their sustainability and versatility in product development.

Moreover, the quest for sustainable food extends beyond ingredient selection to include advancements in food processing technology. Techniques such as pasteurization and heat moisture treatment are being employed to extend the shelf life of products while preserving their sensory attributes and nutritional integrity. These physical and chemical modifications represent a crucial avenue in food technology, contributing to the development of sustainable food options that meet both consumer demands and environmental imperatives.

However, amidst the burgeoning landscape of innovative food products, a pressing need arises to comprehensively understand their physical characteristics. Factors such as texture, viscosity, and structural integrity play pivotal roles not only in enhancing consumer acceptance but also in ensuring the safety, quality, and palatability of these products. Therefore, conducting thorough analyses of the physical properties of innovative foods becomes imperative to effectively position them in the competitive food market.

In essence, the study of physical properties transcends mere academic curiosity; it serves as a cornerstone for unlocking the full potential of innovative food items and fostering a more sustainable and inclusive food system. By bridging the gap between innovation and consumer satisfaction, a deeper understanding of these properties paves the way for a brighter, more sustainable future in food technology.

The presented Special Issue showcases the dynamic landscape of food science, unveiling innovative research across cereals, bioactive components from plants, and meat. These diverse studies underscore the ongoing quest for innovation in food science, offering valuable insights into improving food quality, functionality, and preservation methods to meet evolving consumer demands and industry standards. The total number of submissions to this Special Issue was 15, of which 6 were rejected. The following are the nine accepted and already published papers:

1. Flour Particle Size and Damaged Starch Content: Buresová et al. [1] delve into the impact of botanical origin, flour particle size, and damaged starch content on the characteristics of rice and buckwheat flours. Their findings emphasize the critical role of botanical origin and particle size in determining flour properties, shedding light on the complexities of gluten-free flour’s baking potential.
2. Egg Yolk Granule Functionality: Oladimeji et al. [2] investigate the physical characteristics of egg yolk granules and their functional attributes. Their study underscores the significance of understanding granule properties in enhancing food product development and packaging strategies.
3. Bread Fortification with Wheat Bran and Whey Protein Isolates: Porízka et al. [3] explore the fortification of bread with wheat bran and whey protein isolates, aiming to enhance its nutritional value. Their findings elucidate the effects of fortification on physicochemical and sensory properties, offering insights into improving bread formulations.
4. Heat Treatment Effects on Pork Liver Pâté Quality: Lazárková et al. [4] examine the influence of heat treatment and fat content on pork liver pâté quality. Their study highlights the multifaceted impact of processing parameters on chemical, physical, microbiological, and sensorial properties, essential for optimizing pâté formulations.
5. Techno-Functional Properties of Alternative Plant-Based Flours: Badia-Olmos et al. [5] assess the techno-functional properties of various vegetable flours, unveiling their potential in diversifying plant-based product offerings. Their findings underscore the suitability of alternative flours for developing new food products with distinct properties.
6. Duea Ching Fruit Extract in Sardine Surimi Gel: Buamard et al. [6] investigate the use of Duea Ching fruit extract in sardine surimi gel, exploring its effects on gel properties and storage stability. Their study reveals the potential of the extract in improving gel characteristics and retarding deterioration during storage.
7. Curcumin-Based Emulsion Stability: Opustilová et al. [7] scrutinize the physicochemical properties and stability of multiple curcumin-based emulsions. Their findings elucidate the encapsulation efficiency and stability of curcumin in emulsions, offering insights into potential applications in the food and pharmaceutical industries.
8. Algal Hydrocolloids in Cream Cheese Products: Vincová et al. [8] evaluate the impact of algal hydrocolloids on the physicochemical and textural properties of cream cheese products. Their study provides valuable guidance for optimizing hydrocolloid concentrations to achieve the desired product consistency.
9. Emulsion-Based Coatings for Meat Preservation: Gautam et al. [9] explore the application of emulsion coatings for meat preservation, addressing challenges in maintaining freshness and extending shelf life. Their review underscores the potential of emulsion coatings as sustainable and effective packaging solutions for the meat industry.

In conclusion, the surge in the research and development of innovative and eco-friendly food alternatives reflects a growing commitment to sustainable food production. The influx of plant-based products, such as vegan cheese and high-protein snack bars, underscores a shift towards more environmentally conscious dietary choices. Furthermore, advancements in food processing technology, including techniques like pasteurization and heat moisture treatment, contribute to extending the shelf life of products while preserving their nutritional integrity. However, alongside these innovations, a comprehensive understanding of the physical characteristics of these products is essential. Factors such as texture, viscosity, and structural integrity play crucial roles in ensuring consumer acceptance, safety, and quality. The showcased research in this Special Issue highlights the ongoing quest for innovation in food science, offering valuable insights into improving food quality, functionality, and preservation methods. By bridging the gap between innovation and consumer satisfaction, a deeper understanding of physical properties paves the way for a brighter, more sustainable future in food technology.
